# Translational Genetic Modelling of 3D Craniofacial Dysmorphology: Elaborating the Facial Phenotype of Neurodevelopmental Disorders Through the “Prism” of Schizophrenia

**DOI:** 10.1007/s40473-017-0136-3

**Published:** 2017-11-09

**Authors:** John L. Waddington, Stanislav Katina, Colm M. P. O’Tuathaigh, Adrian W. Bowman

**Affiliations:** 1 Molecular & Cellular Therapeutics, Royal College of Surgeons in Ireland, St. Stephen’s Green, Dublin 2, Ireland; 2 Jiangsu Key Laboratory of Translational Research & Therapy for Neuro-Psychiatric-Disorders and Department of Pharmacology, College of Pharmaceutical Sciences, Soochow University, Suzhou, 215123 China; 3 School of Mathematics and Statistics, University of Glasgow, Glasgow, G12 8QQ UK; 4 Institute of Mathematics and Statistics, Masaryk University, Brno, Czech Republic; 5 Institute of Normal and Pathological Physiology, Slovak Academy of Sciences, Bratislava, Slovakia; 6 School of Medicine, University College Cork, Cork, Ireland

**Keywords:** Neurodevelopmental disorders, Craniofacial dysmorphology, Schizophrenia, Mouse models, 3D facial imaging, Geometric morphometrics, Asymmetry

## Abstract

**Purpose of Review:**

In the context of human developmental conditions, we review the conceptualisation of schizophrenia as a neurodevelopmental disorder, the status of craniofacial dysmorphology as a clinically accessible index of brain dysmorphogenesis, the ability of genetically modified mouse models of craniofacial dysmorphology to inform on the underlying dysmorphogenic process and how geometric morphometric techniques in mutant mice can extend quantitative analysis.

**Recent Findings:**

Mutant mice with disruption of neuregulin-1, a gene associated meta-analytically with risk for schizophrenia, constitute proof-of-concept studies of murine facial dysmorphology in a manner analogous to clinical studies in schizophrenia. Geometric morphometric techniques informed on the topography of facial dysmorphology and identified asymmetry therein.

**Summary:**

Targeted disruption in mice of genes involved in individual components of developmental processes and analysis of resultant facial dysmorphology using geometric morphometrics can inform on mechanisms of dysmorphogenesis at levels of incisiveness not possible in human subjects.

## Introduction

Neurodevelopmental disorders comprise a wide spectrum of human conditions having their origins in disruption to early brain development that can be caused by a variety of genetic abnormalities or environmental adversities. For some, such as trisomy 21 (Down syndrome), 22q11.2 deletion syndrome (velo-cardio-facial syndrome) and foetal alcohol syndrome, their designation reflects a known genetic or environmental cause that indicates a route to investigating the underlying pathobiological process(es). For others, there can be a prolonged process of recognising their sometimes more subtle neurodevelopmental origins before such investigations can begin [1–4].

One of the most consistent anatomical phenotypes of neurodevelopmental disorders is the presence of some form of craniofacial dysmorphology. This relationship, long appreciated and varying from subtle to severe [5, 6], is the inevitable consequence of perturbation to the embryological unity and molecular and physical interplay by which the brain and face develop over early foetal life [7, 8••]. When sufficiently prominent, such dysmorphology can be recognised and categorised qualitatively by inspection, as practiced in clinical genetics and paediatrics, and in the modern era, this is often a prelude to molecular genetic investigation [9]. When less prominent, such dysmorphology can be quantified and graded using traditional anthropometric techniques, i.e. measurements involving standard anatomical landmarks identified on the facial surface of the individual, either manually or via conventional (2-dimensional) photography [10], that can then be subjected to conventional statistical techniques.

However, contemporary approaches increasingly emphasise that craniofacial dysmorphology is best accessed and quantified in its intrinsic 3-dimensional (3D) space. Firstly, this involves 3D acquisition techniques that directly capture the facial surface (i.e. photogrammetry, laser surface imaging) or indirectly allow reconstruction of the facial surface from sequential thin sections across the craniofacies [i.e. X-ray computed tomography (CT), magnetic resonance imaging (MRI)] [6, 11•, 12]. Secondly, such data require a statistical approach, known as geometric morphometrics, that analyses dysmorphology in terms of its size and shape [13]: at a non-technical level, a soccer ball and a table tennis ball have a similar shape but differ in size, while a soccer ball and a rugby ball differ in shape but have a similar size; more technically, “Form is the combination of size and shape of a geometric object in an arbitrary orientation and location. Shape is what remains of the geometry of such an object once you standardize for size” [11•]. These geometric morphometric techniques are applied to 3D images on to which the coordinates of traditional anatomical landmarks can be (a) marked manually, (b) marked automatically, or (c) defined and located from surface curvature [6, 12, 14]; visualisation of the resultant statistical models then reveals their biological import.

Such techniques have been applied to study normal human craniofacial development and dysmorphology not only in recognised neurodevelopmental and other craniofacial disorders [6, 8••, 11•, 15••] but also in normal animals and in putative animal models of human disorders. These typically involve the application of geometric morphometrics to 3D images of the rodent skull and related bony structures, commonly obtained using micro-CT or MRI, on to which traditional anatomical landmarks are applied; this literature is not the topic of the present report and has been reviewed extensively elsewhere [11•, 16–18]. In contrast, very few studies in animal have applied geometric morphometrics to the 3D facial surface which, in contrast to the skull and related bony structures, enjoys the closest embryological intimacy with the brain [7, 8••], in a manner similar to studies in human subjects; in instances where 3D images of facial shape have been obtained in animal models as well as clinically, for example in foetal alcohol syndrome [19], dysmorphology was not analysed using geometric morphometrics. The challenges are accentuated in mice, which are most commonly utilised for gene deletion/transgenic mutation so as to target individual biological mechanisms and create models informative on human genetic disorders.

In this review, we use schizophrenia to illustrate a process of investigation from identification of this illness as a neurodevelopmental disorder characterised by facial dysmorphology, through insights into its genetic origins, to 3D imaging and geometric morphometrics in mice with disruption to a gene associated with risk for schizophrenia, in a manner analogous to clinical studies. There are numerous mutant mouse models related to schizophrenia [20], which have generated an extensive phenotypic database at the levels of behaviour, neurophysiology and brain anatomy. However, facial (as distinct from cranial) dysmorphology in such mouse models has received scant attention due to challenges in acquiring 3D images of the murine facial surface, applying a comprehensive set of landmarks onto those surfaces, and the optimal geometric morphometric approach to analysis thereof. Yet, such investigations in a mutant mouse model would constitute a heuristic for relating facial dysmorphology (the most readily accessible index of brain dysmorphogenesis in human subjects) to abnormal brain structure and function in a manner that is difficult to realise in human subjects. We here describe procedures for conducting such studies and outline initial exploratory findings.

## Schizophrenia: a Neurodevelopmental Disorder with Facial Dysmorphology

Over recent decades, epidemiological evidence and similarly indirect (“soft”) biological findings indicate that schizophrenia is a disorder of subtle neurodevelopmental abnormality [2, 21–23]. However, even the most sophisticated of modern structural and functional neuroimaging techniques applied to young adults with psychotic illness, or even to adolescents showing the putative “at risk mental state”/“attenuated psychosis syndrome”, are unable to resolve hallmarks of abnormal brain development [24, 25]. The need is for a “hard” index of brain dysmorphogenesis during embryonic and foetal life that can be accessed readily in individuals throughout their lifespan.

Congenital anomalies are major malformations that occur to excess in multiple neurodevelopmental disorders, including schizophrenia where their presence is associated prospectively with a doubling of risk for the disorder [26]. Minor physical anomalies (MPAs), including dermatoglyphic and capillary nailfold plexus abnormalities, are more subtle anatomical malformations involving regions of the body that share the ectodermal origins of the brain. However, like congenital anomalies, they occur to excess in multiple neurodevelopmental disorders; hence, their over-representation in schizophrenia [2, 27–31] constitutes a non-specific, qualitative indicator of dysmorphogenesis.

On this basis, we first used anthropometrics to quantify facial dysmorphology in schizophrenia [32], an initiative that continues [33]. Subsequently, we used a subset of linear measures to reconstruct the unique 3D configuration underlying a set of landmarks and applied geometric morphometrics to (a) identify an overall difference in facial shape, but not in size, between schizophrenia cases and controls and (b) resolve in schizophrenia a preliminary 3D topography of subtle, frontonasal craniofacial dysmorphology and asymmetry [34]. We then introduced 3D laser surface imaging technology and automatic application of semi-landmarks over the whole facial surface [35, 36], to resolve the subtle facial dysmorphology of schizophrenia [37] and extended these studies to determine the extent to which facial dysmorphology in schizophrenia is similar to or different from that evident in bipolar disorder [38] and 22q11.2 deletion syndrome [15••], a condition that carries a 25-fold increase in risk for schizophrenia-like psychosis [39].

## Schizophrenia: Exploring Facial Dysmorphology in Mutant Mouse Models

Classical twin and family studies indicate that approximately 70–80% of variation in risk for schizophrenia can be explained by genes, with the number of individual genes associated with that risk continuing to increase [40, 41•]. Neuregulin-1 (NRG1) is a broad family of epidermal growth factors that are associated with various neurodevelopmental and plasticity-related processes [42]. The NRG1 gene has been associated replicably with risk for psychotic illness and with structural and functional neuroimaging abnormalities in psychosis [40, 43, 44]. Thus, members of the NRG1 gene family have been modified in mutant mice to result in a variety of psychosis-related phenotypes [43, 45, 46].

In the work to be outlined here, under approval from the Research Ethics Committee of the Royal College of Surgeons in Ireland, mice with heterozygous deletion of the trans-membrane domain of NRG1 [47] constitute an exemplar for proof-of-concept studies of murine facial dysmorphology in a manner analogous to clinical studies in schizophrenia [37, 48], bipolar disorder [38] and 22q11.2 deletion syndrome [15••].

### Magnetic Resonance Imaging

As described previously [47], eight female NRG1 mutant and eight genetically normal (wild-type, WT) mice were anaesthetised and subjected to MRI at 7 Tesla (Bruker Avance Biospec 70/30 USR, Karlsruhe, Germany). Under anaesthesia, mice were set in a standard position by placing the upper incisors over a conventional “bite bar” and an arm lightly over the dorsal snout; NRG1 mutant and WT mice were imaged alternatively. Two sets of scans were acquired consecutively, during the same session, with settings designed to best capture the brain and craniofacies, respectively: (a) brain: positioned perpendicular to a line connecting the superior end of the olfactory bulb with the superior end of the cerebellum; (b) face: positioned perpendicular to a line connecting the anterior tip of the pinna with the tip of the nose. Three mutually orthogonal (coronal, axial and sagittal) pilot images were used for anatomical orientation of 54 coronal rapid-acquisition relaxation-enhancement images (*TR* = 6300 ms, *TE*_eff_ = 36 ms, RARE factor 8), eight averages (13 min and 26 s total scan time) and field of view 18 mm (matrix 128 × 128 × 54, resolution 0.141 × 0.141 × 0.3 mm^3^). At the end of the experiment, mice were euthanized using an overdose of anaesthetic.

### Anatomical Landmarks and Geometrically Homologous Semi-Landmarks

**Fig. 1:**
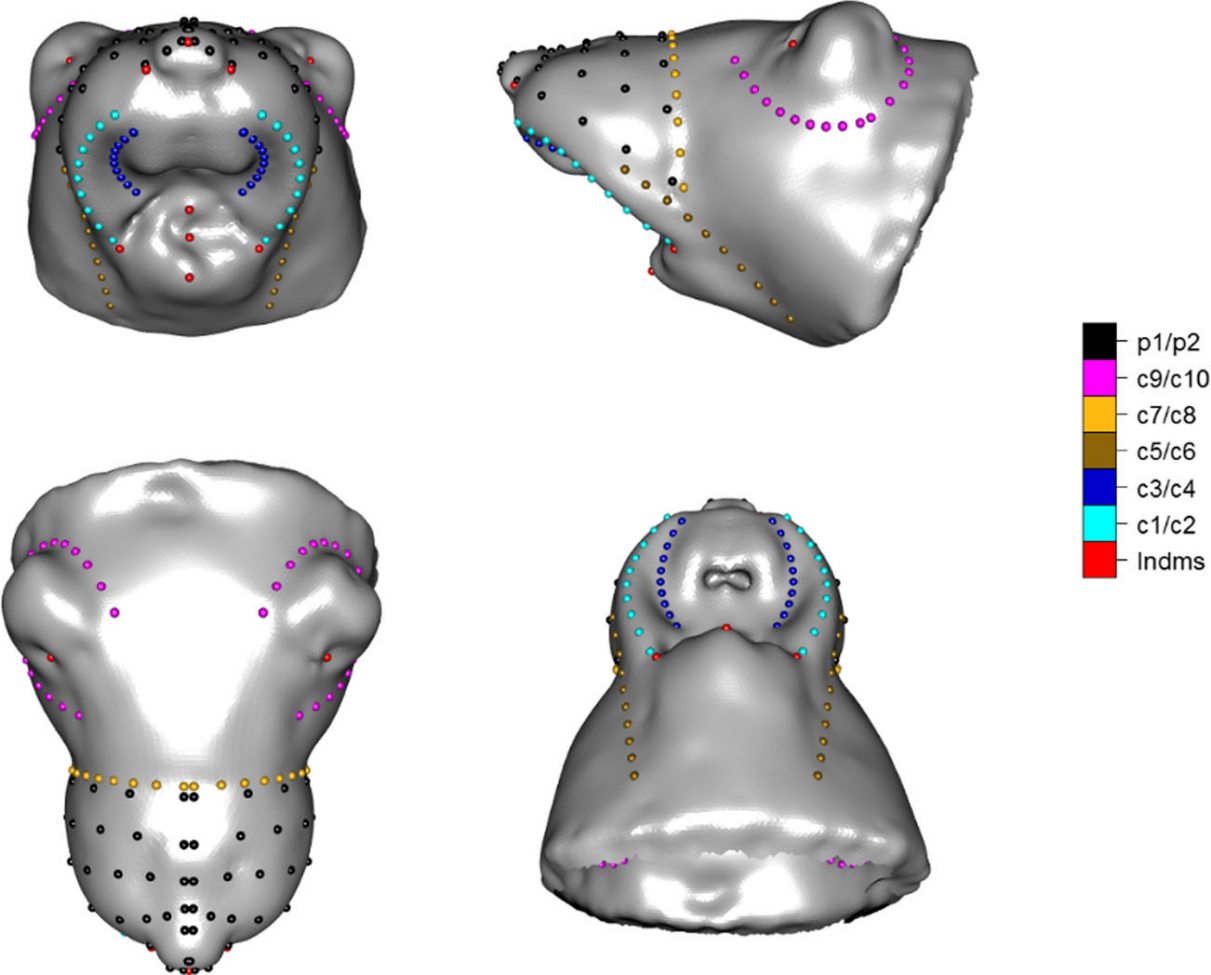
3D design of (semi)landmarks, with anatomical landmarks (lndms), bilateral curves (c1–10; c11/c12 are so adjacent to c9/c10 as to preclude independent visualisation) and patches (p1/p2) on Procrustes mean mouse facial surface (the mean of Procrustes coordinates of all 16 cases and 16 relabelled reflected cases), for each of four views: top left, coronal; top right, sagittal; bottom left, dorsal; bottom right, ventral. In accordance with radiological convention, the left side of the specimen is shown on the right of the image for each view

MRI-derived 3D facial surfaces were reconstructed from coronal MR images using Mimics 9.1 software (Materialise, Leuven, Belgium). Facial shape was characterised first by identifying manually the coordinates of ten biologically homologous anatomical landmarks (s1–10) analogous to those used traditionally in humans: four on the midline and six as left (L) and right (R) counterparts of each of three lateralised points, as illustrated in Fig. 1. These type 2 landmarks, i.e. extremes of curvature (saddle-like points, tip-like points) characterising a single location [12, 15••], were defined as follows:

[s1] *pronasale*: most anterior midpoint of the nasal tip, as tip-like point in cap/peak region; [s2/s3, L/R] *alar curvature* point: point located at the facial insertion of each alar base; following saddle-ridge starting at *pronasale* and continuing to saddle-like point in saddle-ridge region; [s4/s5, L/R] *porion*: highest point on the upper margin of the cutaneous auditory meatus; as deepest point on the front part of the ear lobe, middle point of the entrance to external acoustic meatus and deepest point in a cup/pit region; [s6] *gnathion*: most inferior midpoint on the soft tissue contour of the chin; tip-like point in cap/peak region; [s7/s8, L/R] *cheilion*, L/R: point located at each labial commissure; saddle-like point in saddle-ridge region; [s9] *sublabiale*: most posterior midpoint on the labiomental soft tissue contour that defines the border between the lower lip and the chin; as deepest point in the region between *gnathion* and *labiale inferius*; deepest point in cup/pit region; [s10] *labiale inferius*: midpoint of the vermilion line of the lower lip; as most inferior midpoint on the soft tissue contour of the lower lip; tip-like point in cap/peak region.

From these ten landmarks, 170 geometrically homologous semi-landmarks were positioned along 12 anatomical curves (c1–12), as L/R counterparts of each of six lateralised curves, and across two intervening surface patches [12, 15••]; as illustrated in Fig. 1, this gave a rich characterisation of the craniofacial surface, to include regions where traditional landmarks are not present. Curves can be divided to two types [12, 15••]: (a) valley curves, i.e. a curve following the deepest path in a valley, as defined by points with strongest local positive curvature; (b) ridge curves, i.e. a curve following the ridge, defined by points with strongest local negative curvature:

L/R outer diastemal curve (c1/c2) (ridge curve); L/R outer diastemal curve (c3/c4) (valley curve); L/R dentary curve (c5/c6) (valley curve); L/R zygomatic curve (c7/c8) (valley curve); L/R inferior-ear curve (c9/c10) (valley curve); L/R posterior-ear curve (c11/c12) (valley curve). In Fig. 1, (c9/c10) and (c11/c12) in effect jointly create one continuous valley that is rendered visible as an ear curve (c9/c10) (valley curve); p1/p2 is a closed surface patch between four curves (c1/c2, c7/c8) and three landmarks (s1–3) that are used for warping purposes in the course of geometric morphometric analysis (see succeeding texts).

Semi-landmarks were initially positioned on anatomical curves and intervening surface patches in an equidistant manner. The semi-landmarks were subsequently repositioned by sliding across the curves and surfaces, using the minimisation of bending energy (BE) [12, 15••] as a criterion, to create geometrically homologous points with respect to a symmetrised reference shape. The equidistant resampling and sliding of semi-landmarks and subsequent statistical analyses were performed in R software system [49].

### Geometric Morphometrics and Visualisation

Firstly, one particular facial image was chosen as a reference shape, symmetrised, to create a template for visualisation. Secondly, Generalised Procrustes Analysis (GPA) [13] was performed to remove effects of location, scale and orientation and to calculate the Procrustes mean shape. This was then symmetrised, to provide a new reference shape, and sliding was repeated using this reference. These two steps, GPA and sliding, were repeated iteratively until convergence (in total, five iteration steps). For final registration of the images, shape coordinates were calculated by GPA.

For visualisation, all surface points were estimated by interpolating thin-plate spline (TPS) based on 180 landmarks and semi-landmarks, using the first craniofacial triangulated surface as reference. To test overall group differences, a permutation Goodall *F*-test (999 permutations) [13, 15••] was used; *p* values were calculated for the entire semi-landmark set, each curve and surface patch separately. For visualisation, *F*-statistics were calculated point-wise and displayed as coloured surface maps (statistical parametric map, SPM) based on 27,490 point locations. To explore the nature of difference between the groups, the linear discriminant analysis (LDA) was used. The results of LDA (in LD_1_ subspace; since there are only two groups, WT and NRG1, there is only LD_1_) were visualised by SPMs of 27,490 point-wise Procrustes shape distances (PSDs) calculated between two extreme shapes estimated in the LD_1_ subspace.

Asymmetry was quantified by comparing the landmarks and semi-landmarks with reflected and relabelled (RR) versions constructed with respect to the mid-sagittal plane. Firstly, this plane was estimated from four unpaired mid-sagittal landmarks (s1, s6, s9 and s10) by ordinary least squares and rotated to correspond to the (*x*,*y*) plane. Secondly, the sign and labels of paired landmarks and semi-landmarks were reversed across L and R sides of the craniofacial surface. The original Procrustes shape coordinates (PSC), together with their RR counterparts, were jointly submitted to GPA to register both into the same shape space. Fluctuating asymmetry (FA) expresses how the difference between the original and RR shapes fluctuates in the sample; this is calculated as the sum of squares of individual asymmetry scores, i.e. Procrustes distances between original and RR PSC of each shape. The asymmetry of the means (AM) is calculated as the sum of squares of Procrustes distances between the original and RR Procrustes mean shape and, when multiplied by sample size, is called directional asymmetry (DA).

To test DA, we used a permutation Mardia-Bookstein *F*-test (999 permutations) [15••, 50]. Probability (*p*) values were calculated for the complete semi-landmark set, each curve and surface patch separately. All permutation two-sample *t*-tests (999 permutations) for mean difference were performed in the Procrustes shape space of both WT and NRG1 mutants, and also in *separate* subspaces of WT and NRG1 mutants. To test AM and DA, PSD and BE approaches can be used; both are independent of shape orientation but dependent on mean shape choice. To explore the nature of differences in asymmetry between the groups, we again used LDA in LD_1_ subspace, visualised by SPMs of 27,490 PSDs calculated between two extreme shapes estimated in LD_1_ subspace of asymmetry.

## Facial Dysmorphology in NRG1 Mutant vs WT Mice

### Overview

In initial exploratory analyses, while overall shape did not differ between NRG1 mutants and WT across the whole semi-landmark set (Goodall *F*-test, *p* = 0.4), there were marginal differences for the dentary curves (c5/c6, *p* = 0.07) and the outer diastemal curves (c3/c4, *p* = 0.09) but not for other curves or surface patches (*p* > 0.2).

### Facial Dysmorphology

**Fig. 2:**
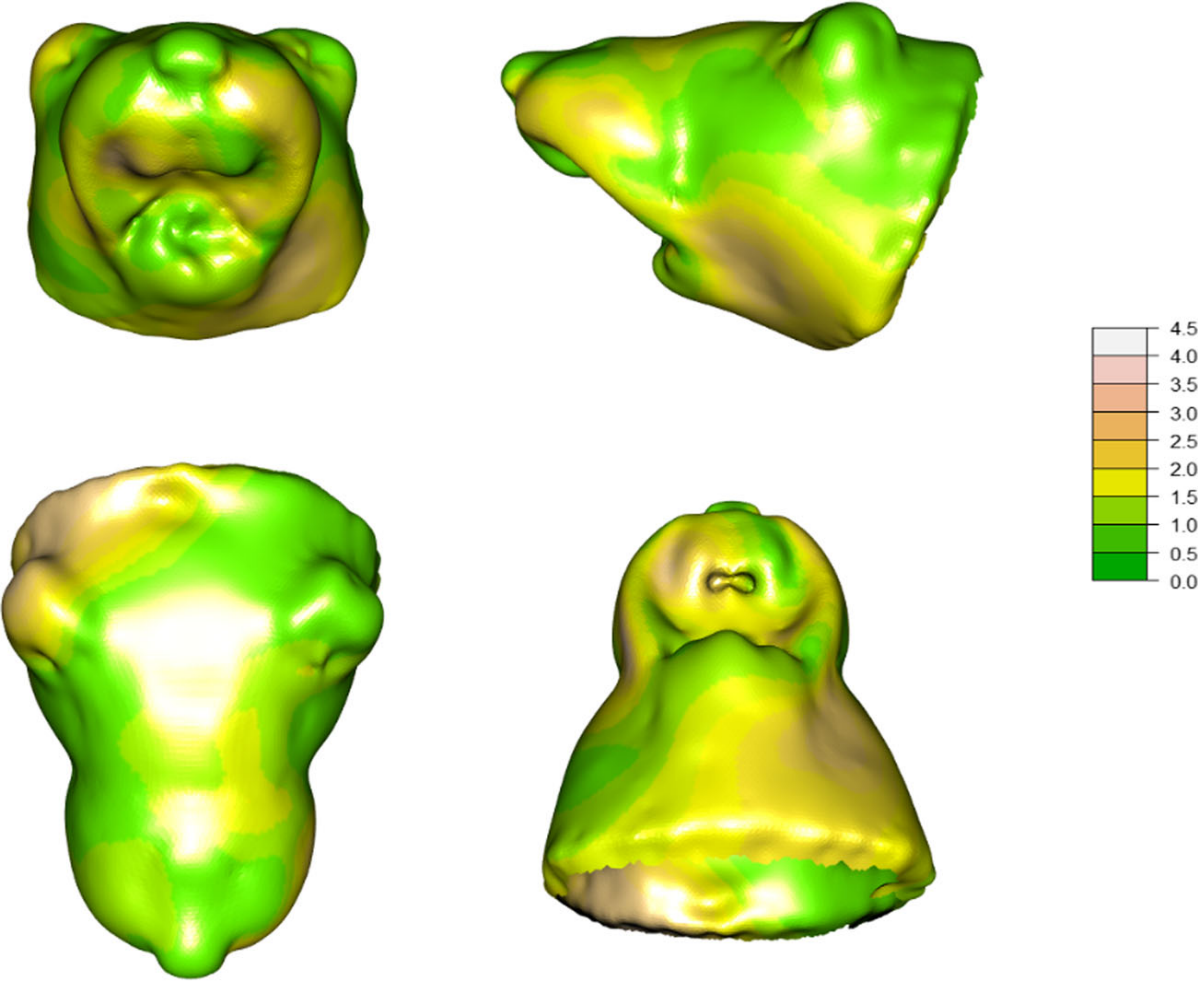
Statistical parametric maps of *F*-statistics for differences between NRG1 and WT across the 27,490 point locations on the facial surface, for each of four views: top left, coronal; top right, sagittal; bottom left, dorsal; bottom right, ventral. The scale indicates values of *F*-statistics, with values of 1.743 and above indicating significance at *p* ≤ 0.05. In accordance with radiological convention, the left side of the specimen is shown on the right of the image for each view

Figure 2 shows a SPM for comparisons between NRG1 and WT across the 27,490 point locations on the facial surface; in accordance with radiological convention, the left sides of the specimen are shown on the right of the images as viewed. The scale in Fig. 2 indicates values of *F*-statistics, with values of 1.743 and above indicating significance at significance level *p* = 0.05 (based on *F*-distribution with 14 and 196 degrees of freedom; see [50]): differences between NRG1 and WT were most evident (i.e. orange to pink-grey) for the left anterior-dorsal/ventral maxillary and left posterior-ventral regions of the snout, the right anterior-ventral maxillary region of the snout and a right posterior-dorsal periauricular region; the anterior mandibular and other regions of the snout and head (i.e. green) did not differ systematically between NRG1 and WT.

**Fig. 3:**
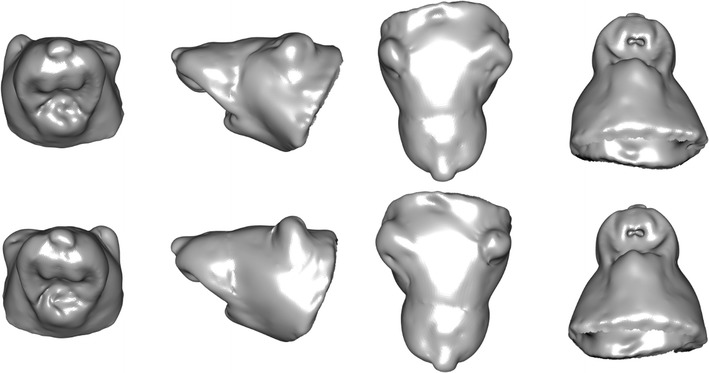
3D visualisation as plain surfaces for (top row) extreme WT shape and (bottom row) extreme NRG1 shape of LDA of differences in asymmetry in LD_1_ subspace (after arbitrary magnification) for each of four views: left, coronal; centre-left, sagittal; centre-right, dorsal; right, ventral. In accordance with radiological convention, the left side of the specimen is shown on the right of the image for each view

### Asymmetry

Directional asymmetry (the same as AM at a significance level *p* = 0.05) was present in both WT (whole semi-landmark set, *p* < 0.001) and NRG1 mutants (whole semi-landmark set, *p* < 0.001). Overall, the evidence for differences in DA between NRG1 mutants and WT across the whole semi-landmark set (*p* = 0.1) is not convincing, but there were significant differences for the inferior ear curve (c9 vs c10, *p* = 0.04) and marginally for the posterior ear curve (c11 vs c12, *p* = 0.06). The overall mean asymmetry ratio (*FA*_PSD_ NRG1/WT) was 1.76, indicating greater asymmetry in the NRG1 group; *FA*_PSD_ was highest for the inferior ear curve (c9/c10, *FA*_PSD_ = 3.22) and the posterior ear curve (c11/c12, *FA*_PSD_ = 2.04), i.e. the curves showing the most statistically robust differences in DA between NRG1 mutants and WT. Figure 3 shows plain surfaces for extreme NRG1 shape and extreme WT shape of LDA of differences in asymmetry in LD1 subspace; in accordance with radiological convention, the left sides of the specimen are shown on the right of the images as viewed.

## Discussion

### Overview

In this report, we consider (a) the recognition and study of facial dysmorphology in schizophrenia as an externally accessible index of brain dysmorphogenesis that can be more easily evaluated in clinical populations than can the brain itself and (b) the quantitative assessment of craniofacial dysmorphology in a genetically modified (and, by extension, in any early adversity-determined) mouse model of schizophrenia in a manner analogous to clinical studies of 3D facial dysmorphology in schizophrenia and its 22q11.2DS human genetic model [15••, 37, 38].

### Facial and Brain Dysmorphogenesis

Mice with disruption of NRG1, a gene associated meta-analytically with risk for schizophrenia [40, 44], were studied on a proof-of-concept basis. As expected, geometric morphometric analysis revealed no systematic facial dysmorphology in NRG1 mutants at a global level. However, at a local level, there is some evidence for dysmorphology in the left anterior-dorsal/ventral maxillary and left posterior-ventral regions of the snout, the right anterior-ventral maxillary region of the snout, and a right posterior-dorsal periauricular region. The anterior mandibular and other regions of the snout and head were largely unaffected. It is unreasonable to expect any simple concordance between the topographies of facial dysmorphology in schizophrenia patients and in mice with disruption of a single risk gene among the large number of genes associated with risk for the disorder [40, 41•, 44]; the greater import is a research paradigm and analytical approach that is capable of resolving a topography of facial dysmorphology in mice with disruption of a given risk gene for schizophrenia and can be generalised across neurodevelopmental and other craniofacial disorders.

The complex cellular processes that underlie the embryological unity and molecular and physical interplay by which the face and brain develop over early foetal life are increasingly understood and appear to generalise across mammalian species but are beyond the scope of the present report (see [8••, 11•]). Craniofacial anomalies can largely be traced to abnormalities in the formation, migration and differentiation of neural crest cells [8••, 51]. That NRG1 and its erbB4 receptor have been shown to play an important role in the development of ectodermal/neural tissue, including mouse craniofacies [42, 52, 53], suggests a putative basis for the facial phenotype of NRG1 mutant mice.

### Facial and Brain Asymmetry

A notable feature is the presence of asymmetry in mouse facial morphology and its disruption in NRG1 mutants. Asymmetry is an intrinsic feature of the human face that influences normal human behaviours such as perception of attractiveness and is disrupted in neurodevelopmental conditions such as autism spectrum disorder [54–56]; similarly, the normal human brain is asymmetric in a manner that may be disrupted in schizophrenia [57, 58•]. Asymmetry in the mouse craniofacies [59] and brain [60] is also recognised. While craniofacial asymmetry in mice can be altered by foetal exposure to alcohol or other dysmorphogens, this is typically assessed using geometric morphometrics of skeletal landmarks, linear distances, or qualitative evaluation of the facial surface [19, 61]. Geometric morphometric analysis of the facial surface indicated that while both NRG1 mutant and WT mice showed facial asymmetry, this was accentuated in NRG1 mutants.

The complex cellular processes that underlie the development of asymmetry are also increasingly understood and appear to generalise across mammalian species, but they are again beyond the scope of the present report (see [62, 63]). Among these, nodal, an evolutionarily conserved member of the transforming growth factor-β (TGF-β) superfamily of secreted signalling factors, plays a key role in embryonic development and asymmetry [64–66]. That NRG1 and TGF-β signalling cascades appear to overlap in promoting cellular differentiation and migration [67] suggests a putative basis for the asymmetric facial phenotype of NRG1 mutant mice.

## Conclusions and Future Directions

In the context of a panoply of human developmental conditions, the studies reviewed here indicate the following: (a) the conceptualisation of schizophrenia and related psychotic illness as neurodevelopmental disorders; (b) the status of craniofacial dysmorphology as a clinically accessible index of brain dysmorphogenesis; (c) the ability of genetically modified mouse models of craniofacial dysmorphology to inform on the underlying dysmorphogenic process; (d) how geometric morphometric techniques in mutant mice can extend quantitative analysis of dysmorphology from the skull and related bony structures to the ectodermally derived facial surface that enjoys the greatest embryological intimacy with the brain; and (e) how targeted disruption in mice of genes involved in individual components of the dysmorphogenic process can inform on pathobiology at levels of incisiveness not possible in human subjects.

Going forward, studies in mutant (and, indeed, in any early adversity-determined) mouse models should recognise and confront a number of challenges: Firstly, facial shape is well-recognised to differ between men and women, with aspects of dysmorphology in human developmental disorders differing between male and female subjects [6, 55]; hence, studies in mice should involve systematic comparisons between the sexes. Secondly, human facial dysmorphology can be potentially modified by factors unrelated to dysmorphogenesis, such as disease-related weight loss or drug treatments associated with weight gain [37, 38]; hence, studies in mice should clarify any influence of these factors. Thirdly, evolving understanding of (a) genetic regulation both of normal craniofacial morphology and of risk for human neurodevelopmental disorders and (b) gene expression domains in distinct fields of craniofacial development [7, 8••, 11•] behoves investigators to exploit the full potential of these insights in future mouse models.
